# Quantification of Extracellular Volume in CT in Neoadjuvant Chemotherapy in Breast Cancer: New Frontiers in Assessing the Cardiotoxicity of Anthracyclines and Trastuzumab

**DOI:** 10.3390/jpm13020199

**Published:** 2023-01-22

**Authors:** Marcello Chiocchi, Martina Cerocchi, Federica Di Tosto, Roberto Rosenfeld, Monia Pasqualetto, Gianluca Vanni, Vincenzo De Stasio, Luca Pugliese, Carlo Di Donna, Gaetano Idone, Saverio Muscoli, Ilaria Portarena, Mario Roselli, Francesco Garaci, Roberto Floris

**Affiliations:** 1 Department of Biomedicine and Prevention, Division of Diagnostic Imaging, University of Rome “Tor Vergata”, 00133 Rome, Italy; 2 Department of Diagnostic Imaging and Interventional Radiology, Ospedale Fatebenefratelli Isola Tiberina-Gemelli Isola, 00186 Rome, Italy; 3 Medical Oncology Unit, Department of Systems Medicine, University of Rome “Tor Vergata”, 00133 Rome, Italy; 4 Breast Unit, Department of Surgical Science, University of Rome “Tor Vergata”, 00133 Rome, Italy; 5 Unit of Cardiology, Department of Systems Medicine, University of Rome “Tor Vergata”, 00133 Rome, Italy

**Keywords:** extracellular volume, cardiotoxicity, chemotherapy

## Abstract

Breast cancer patients undergoing neoadjuvant chemotherapy with anthracyclines or trastuzumab can suffer cardiotoxic issues. Nowadays, the markers of cardiac damage are still not reliable, and extracellular volume (ECV) calculated from CT could be a promising cardiotoxic marker. Eighty-two patients, treated with two different chemotherapy regimens based on doxorubicin (DOX) or epirubicin-trastuzumab (EPI–TRAS), were retrospectively selected and the variations in extracellular volume (ECV) values were measured and analyzed. Whole Body CT (WB-CT) scans were acquired after 1 min, in the portal phase (PP), and after 5 min, in the delayed phases (DP), at the baseline (T_0_), after one year (T_1_) and after five years (T_5_) from the end of chemotherapies. The values measured by two radiologists with different levels of experience were evaluated in order to assess the inter-reader reproducibility assessment (ICC = 0.52 for PP and DP). Further, we performed a population-based analysis and a drug-oriented subgroup analysis in 54 DOX-treated and 28 EPI–TRAS-treated patients. In the general cohort of women treated with any of the two drugs, we observed in the lapse T_0_–T_1_ a relative increase (RI) of 25% vs. 20% (PP vs. DP, *p* < 0.001) as well as in the lapse T_0_–T_5_ an RI of 17% vs. 15% (PP vs. DP, *p* < 0.01). The DOX-treated patients reported in the lapse T_0_–T_1_ an RI of 22% (*p* < 0.0001) in PP and an RI of 16% (*p* = 0.018) in the DP, with ECV values remaining stably high at T_5_ both in PP (RI 14.0%, *p* < 0.0001) and in DP (RI 17%, *p* = 0.005) highlighting a possible hallmark of a persisting CTX sub-damage. On the other hand, ECV measured in EPI–TRAS-treated women showed an RI in T_0_–T_1_ of 18% (*p* = 0.001) and 29% (*p* = 0.006) in PP and DP, respectively, but the values returned to basal levels in T_5_ both in the PP (*p* = 0.12) and in DP setting (*p* = 0.13), suggesting damage in the first-year post-treatment and a possible recovery over time. For the 82 patients, an echocardiography was performed at T_0_, T_1_= 12 m + 3 m and T_5_ = 60 m + 6 m with LVEF values at T_0_ (64% ± 5%), T_1_ (54% ± 6%) and T_5_ (53% ± 8%). WB-CT-derived ECV values could provide a valid imaging marker for the early diagnosis of cardiotoxic damage in BC patients undergoing oncological treatments. We detected different patterns during the follow-up, with stably high values for DOX, whereas EPI–TRAS showed a peak within the first year, suggesting different mechanisms of cardiac damage.

## 1. Introduction

In the era pre-COVID-19, cardiovascular disease and cancer diseases were the most common causes of death in the world and, in particular, in the middle-upper and higher income countries [1,2].

It has been estimated that a woman, without additional genetic factors, has a lifetime risk of one in eight (about 12.4%) to receive a diagnosis of breast cancer (BC) [3], with a five-year survival rate of about 80% [4].

The main adjuvant and neoadjuvant chemotherapy regimens involve [5] the use of anthracyclines (AC), although recently new AC-free schemes are recommended for Her-2 positive BC [6,7] and in postmenopausal women presenting with a luminal-like lobular histotype where an endocrine therapy is preferred [8,9]. Indeed, AC and trastuzumab are among the most efficient antineoplastic drugs, particularly in triple negative BC (TNBC) and HER-2 positive BC, respectively, but they are burdened with an important cardiotoxicity (CTX) rate [10,11], especially doxorubicin (DOX) [12]. The main cardiac insult is expressed as a progressive increase in myocardial fibrosis [13], clinically ranging from left ventricular ejection fraction (LVEF) depression to symptomatic heart failure [12]. Therefore, the assessment of cardiac function before, during and after treatment is a medical necessity for these patients in order to prevent the onset of CTX, limit its incidence and regulate anti-tumor therapy [14]. Ventricular function may be studied through echocardiography calculation by the measurement of the ejection fraction (EF). However, the EF, initially considered the gold standard, has a suboptimal reproducibility, is an operator-dependent examination and is not able to reflect a regional function, only a global one. Moreover, the decrease in left ventricular ejection fraction seems to be a delayed process in the evolution of the disease [15,16,17]. The myocardial extracellular volume (mECV), classically measured in MRI, is used as imaging marker which can be considered in the diagnosis of cardiac fibrosis [18], reflecting the loss of myocardiocytes [19,20]. However, the limited availability and high cost of MRI limits its routinary use in cardio-oncology [19]. Therefore, to overcome these limitations, the calculation of ECV by computed tomography (CT) has been recently proposed [21]. CT-derived ECV has proven to have a high correlation with both MRI-derived ECV and histological findings [13,21,22,23,24].

The aim of this study was (i) to investigate a possible validation of myocardial ECV measured from total body CT as an early imaging marker of myocardial damage, evaluating whether neoadjuvant chemotherapy (NACT) in BC patients is associated with an increase in ECV value even in the absence of clinical, echocardiographic or electrocardiographic signs of cardiac damage; (ii) to analyze ECV values one year after the end of treatment and five years later with the same objective; (iii) to evaluate whether ECV values in patients who received Adriamycin as chemotherapy are different from those who received epirubicin and trastuzumab.

## 2. Materials and Methods

### 2.1. Study Design

We retrospectively selected 102 whole body computed tomography (WBCT) about female patients affected by BC who underwent neoadjuvant chemotherapy (NACT) with an anthracycline as backbone therapy between January 2010 and July 2016 at Tor Vergata University Hospital, Rome, Italy. All patients’ heart condition and oncological status was assessed at baseline (T_0_), at the first-year post-therapy (T_1_) and at the fifth-year post-therapy (T_5_) during the oncological follow-up.

In the study were included adult females (age > 18 years) with a histological diagnosis of BC and with a therapeutic decision to perform NACT, without any further restrictions.

Exclusion criteria were as follows: (i) the presence of a pre-existing cardiac disease; (ii) previous cardiotoxic treatments, in particular chemotherapeutic treatments, and chest irradiation therapies (women treated for a left BC or mediastinal lymphomas); (iii) hematocrit examinations performed outside the indicated time interval (4 weeks before or after the CT examination); (iv) cases in which chemotherapy dose reduction was necessary; and (v) patients with a relapse or metastases from BC or a second tumor who underwent to additional oncological treatment.

### 2.2. Chemotherapeutic Schemes Used

The patients were treated with polychemotherapeutic protocols with a neoadjuvant purpose. The schemes used were AC-based using either DOX (cumulative dose 240 mg/m^2^) or epirubicin (EPI, cumulative dose 360 mg/m^2^) following the guidelines for locally advanced BC. Patients with BC were considered for treatment schemes, such as AC-TXT (doxorubicin 60 mg/mq, Cyclophosphamide 600 mg/mq, IV, g1 q21 for 4 cycles, then Docetaxel, 100 mg/mq, IV, each 21 days, for 4 cycles) and FEC (5-Fluorouracil 500 mg/mq, epirubicin 100 mg/mq, Cyclophosphamide 500 mg/mq, IV, each 21 days, for 3 cycles), alternatively. In Her-2 positive BC patients, trastuzumab (TRAS) was delivered weekly during association with FEC scheme (8 mg/Kg as first load dose, then 6 mg/Kg, g1, each 21 days for 4 cycles, then 14 cycles of maintenance delivered at 6 mg/Kg, every 3 weeks). Chemotherapy doses were adjusted for body weight and body surface area according to clinical guidelines.

### 2.3. CT Acquisition Protocols

The total-body CT scans, often required in patients with BC for staging or other clinical reasons, were performed with a 128-layer CT scanner (Revolution EVO; GE-Healthcare; Revolution EVO, CT, General Electrics Medical System, Milwaukee, WI, USA) using a multi-step spiral acquisition and with cranial–caudal scan direction. The scans included the abdomen, part of the thorax, the heart and the skull. The acquisitions were performed during scheduled oncologic follow-ups; therefore, without ECG gating. The imaging acquisition adhered to a standard protocol requiring a baseline scan and three subsequent scans after administration of 100–120 mL of iodinated contrast (Iomeron 350 mg/mL, Bracco Imaging) followed by 30–50 mL of saline (injection rate of 3 mL/s).

The post-contrast scans were acquired applying the bolus tracking technique: a threshold of 120 HU was set to start the scan using a region of interest (ROI) placed in the descending aorta at the thoracic-abdominal passage. Three phases were obtained:

- Arterial phase, generally about 15–18 s after contrast injection;
- Portal phase, 1 min after contrast injection;
- Delayed phase, about 5 min after contrast injection.

Radiation doses were reported using the following formula: dose length product (DLP) expressed in mGy × cm (DLP value for each patient were extracted).

### 2.4. CT Image Analysis

WB-CT images were retrospectively evaluated by two radiologists with at least 5 years of experience in cardiovascular radiology. Each operator chose the best slice to visualize an axial view of the four cardiac chambers.

The regions of interest were placed in the interventricular septum and in the ‘blood’ pool of the left ventricle and they were measured in PP and DP, respectively, drawing them of equal proportions.

ROIs were placed in the thickest portion of the middle septum, excluding areas closer to the ventricular cavity to avoid blood contamination. Moreover, papillary muscles were avoided.

**Figure 1:**
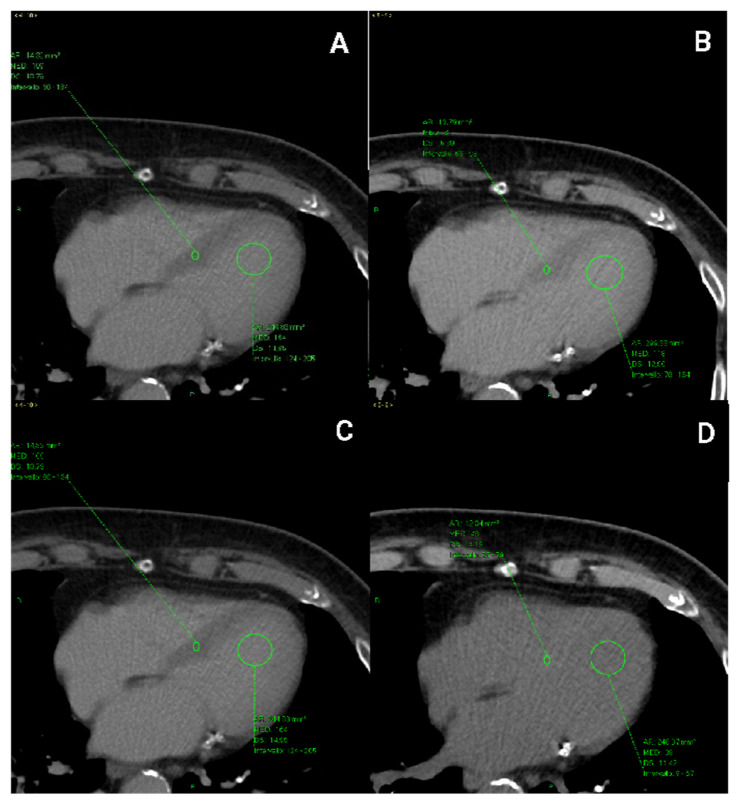
ROI (green arrows) located in the interventricular septum and left intraventricular blood pool in CT scans acquired (**A**) during the portal phase (1 min after contrast mean injection) and (**B**) in the delayed phase (5 min after contrast mean injection), respectively. ROIs used for ECV calculation in CT scans were acquired (**C**) in the pre-contrast phase, where the ventricular septum appears more hyperdense than the cardiac chambers (**D**) and in the portal phase.

In the pre-contrast scans, the ROIs were positioned at the same level chosen in the post-contrast scans (at 1 min and 5 min). ROIs were measured in the three phases (basal, portal, and delayed phase) at time 0, within one year and after five years in order to obtain Hounsfield units (HUs) to include in the ECV calculation of the portal and delayed phase for each available examination (Figure 1).

ECV was determined by the following equation, according to Miller et al. [25].

ECV = (1 − *haematocrit*)∙[(HU*myo*_post_ − HU*myo*_pre_)/(HU*blood*_post_ − HU *blood*_pre_)].

Standard deviations of ROIs were calculated to minimize the interference of motion artefacts on the myocardial HU measurements. Therefore, altered values were not considered in this study.

### 2.5. Participant Selection and Study Population

**Figure 2:**
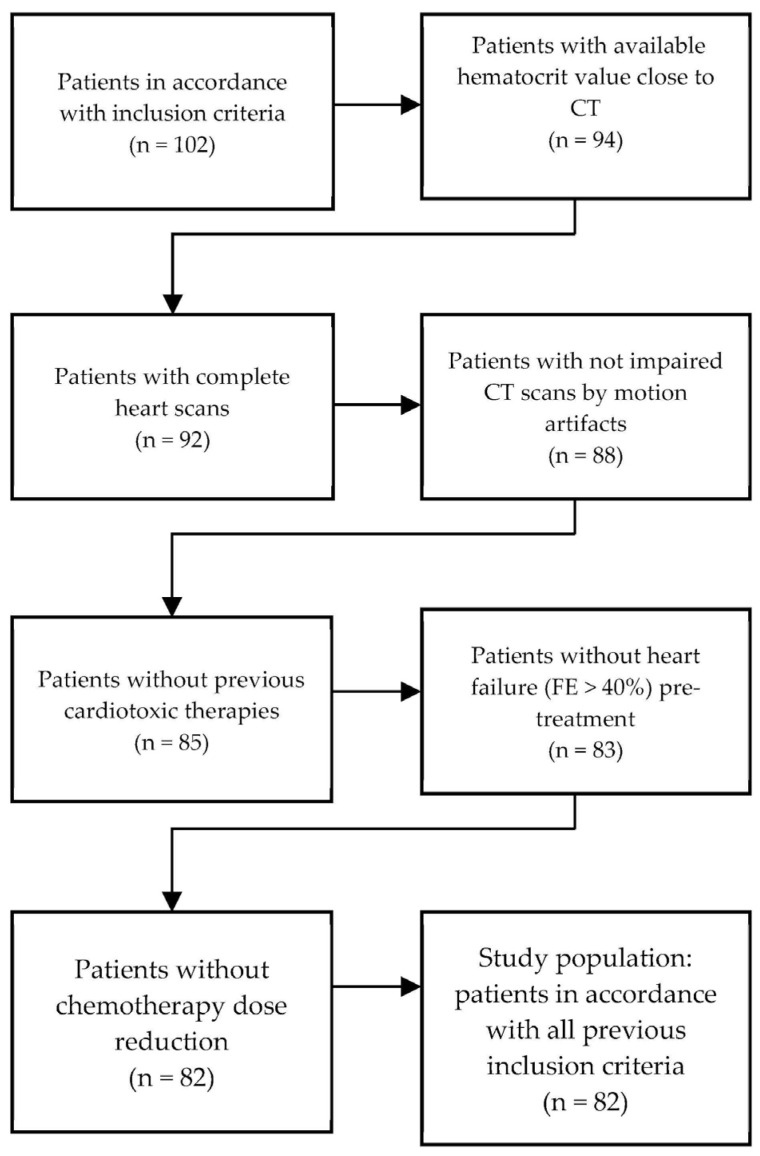
Selection of the study population.

From the preliminary selected population (*n* = 102), 8 patients were excluded because the hematocrit examinations were performed outside the indicated time interval (more than 4 weeks before or after the CT examination), 2 patients because the CT scans were incomplete, 4 patients because the CT images were compromised by motion artefacts, 2 patients who had already received radiotherapy for left breast cancer, 1 patient who had already received another cardiotoxic chemotherapy, 2 patients who had a previous heart disease (FE < 40%) and 1 patient because the chemotherapy dose had to be reduced to 75% during treatment due to febrile neutropenia. After applying the exclusion criteria, the population considered for the final analyses accounted for 82 patients (Figure 2 and Table 1).

At the time of diagnosis, the population median age was 53 years (IQR 47.5–58.5), with 62 patients affected by ductal carcinoma, 4 patients by infiltrating lobular carcinoma, 14 patients by a poorly differentiated carcinoma and 2 patients by an undifferentiated carcinoma. About 44 patients reported a stage II tumor (following TNM 8th edition), 16 presented with a stage IV and 14 stage III and 8 stage Ic (24). Of the patients, 54 were treated with doxorubicin (DOX)-based treatment and 28 with a epirubicin–trastuzumab (EPI–TRAS) based treatment. All patients were healthy at T_0_, with hematocrit and LVEF mean values of 38.8% ± 4% and 64% ± 5%, respectively (Table 1). At the ECV follow-up after 5 years, all 82 patients were considered, as they adequately fit the criteria.

### 2.6. Statistical Analysis

The statistical analysis and plots were performed with RStudio software (v 4.0.4; source http://www.R-project.org (accessed on 3 October 2022)). Data are presented as mean ± standard deviation (SD) for normally distributed populations and median ± interquartile range (IQR) for not normally distributed ones. When not otherwise specified in the text, we referred as the mean ± SD. Shapiro–wilk tests were used in order to assess the normality of the curves. For multiple comparisons ANOVA test was chosen after carrying Shapiro–wilk and Mauchly’s test in order to assess normality and sphericity, respectively. Afterward, a pairwise test was conducted only for the most important post hoc analysis through t-student test for paired data. In the case of different, independent groups with different or not known variances, a Welch’s test was performed, whereas, for non-normal distributions, a Wilcoxon test for paired data was used. All the tests were performed with a two-sided α-value = 0.05 as significance level. Eventually, the inter-class correlation (ICC) function was used in order to assess inter-observer reproducibility [26].

## 3. Results

### 3.1. Inter-Observer Differences and Reproducibility between Operators

**Figure 3:**
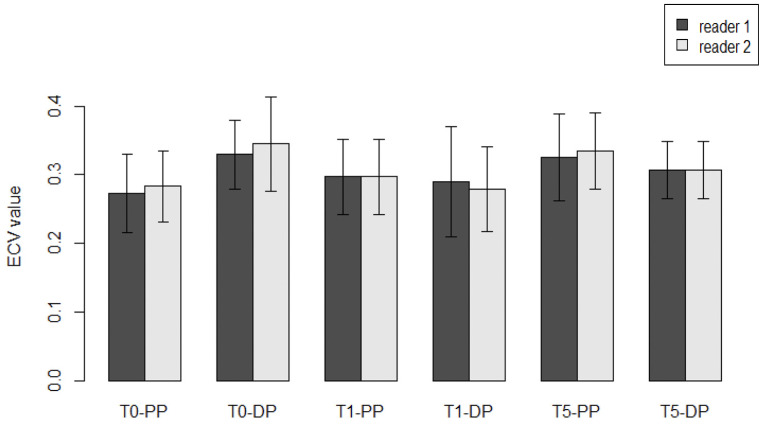
Comparison between two readers with different experience. The bar plots show the mean values for ECV measurements between two readers during FU in different moments, at the baseline, at the first year and at the fifth year, both in the portal phase (PP) and delayed phase (DP). All the comparisons were not significative (*p* > 0.05).

Data for the same patients were collected separately from two different operators blinded from each other, avoiding performance biases, and with at least 5 years of experience in cardiovascular radiology. DLP and SD were measured by the two operators in all 82 WB-CT scans, while the inter-observer variability was minimized including the same volumes. The mean value and SD detected were 3114 ± 9 mGy × cm, with a minimum DLP value of 2797 mGy × cm and a maximum value of 3849 mGy × cm. Therefore, an ICC analysis was conducted to assess the reliability and reproducibility of the procedure. ECV mean values and SD are reported in Table 2 and Figure 3.

The pre-treatment ECV (T_0_) measured in the portal phase (PP) was 27% ± 6% vs. 29% ± 8% (median + IQR), as rated by the first and second reader, respectively, with a non-significant difference (*p* = 0.09). In the delayed phase (DP), ECV was detected by first and second reader to be 28.3% ± 5% vs. 27.9 ± 6% (mean + SD), respectively, with a non-significant difference (*p* = 0.64). Similar results were observed when the two raters were compared at the first year of FU (T_1_) both for portal phase (32.6% ± 6% vs. 32.9% ± 5%, *p* = 0.77) and delayed phase (34.5% ± 7% vs. 33.5% ± 6%, *p* = 0.47). At the fifth-year checkpoint (T_5_), no significant differences were found between the two readers, neither in the portal phase (30.0 ± 5% vs. 31.0 ± 7%, *p* = 0.68), nor in the delayed phase (32.7% ± 7% vs. 31.2% ± 7%, *p* = 0.34), respectively.

Furthermore, an interclass correlation (ICC) analysis was conducted with the aim to assess the agreement and reproducibility of the inter-observer tests. For this purpose, a random sample of 10 subjects was chosen from the two observer’s rating groups in order to limit the weight of systematic and random biases. An ICC coefficient of 0.514 (95% CI 0.201–0.733) and 0.518 (95% CI 0.201–0.737) for the portal phase and the delayed phase, respectively, revealing an agreement between the two observers.

### 3.2. Results for the General Population of Women Treated with Cardiotoxic NACT

In order to establish the validity of ECV measurements in WB-CT, a total of 82 patients who underwent cardiotoxic NACT were evaluated. ECV was measured at three different checkpoint times: at baseline (T_0_), the first year after NACT termination (T_1_) and the fifth year thereafter (T_5_). The population analyzed was heterogeneously represented by DOX-based schemes (DOX) and the combination of epirubicin and trastuzumab (EPI–TRAS), in order to combine information and assessing ECV validity in a general population. Further analyses were performed in the two subgroups with the aim to highlight different trends between them.

Concerning the further analyses, all the measurements were considered only for the more experienced observer.

#### 3.2.1. ECV Measured in the General Population in the Portal Phase (PP) Setting

**Figure 4:**
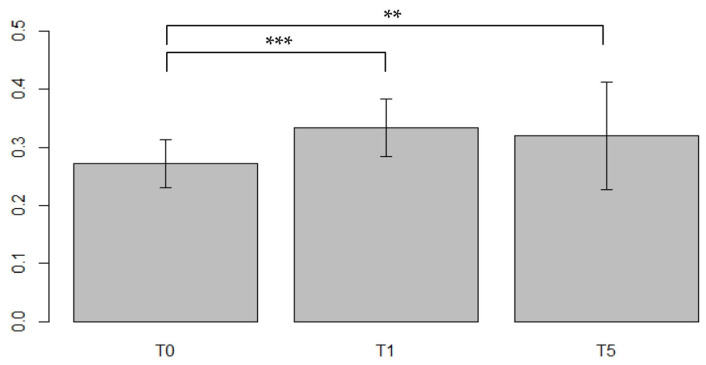
Comparisons of the ECV values from the same population between different timepoints in the Portal Phase. ECV values measured at baseline (T_0_), after first year post-treatment (T_1_) and after 5 years post-treatment (T_5_). strongly significant values were associated within the comparison T_0_–T_1_ (*p* < 0.0001) suggesting a strong interaction between CTX drugs and myocardial precocious damage, confirmed also after 5 years with stable high values, significant as well (*p* < 0.01). *: barely significant, **: significant, ***: strongly significant.

A significant difference in ECV values was found between T_0_–T_1_ with an increasing of 25% (V = 73, *p* < 0.0001) and between T_0_–T_5_ with a relative increase (RI) of 17.5% (V = 224, *p* = 0.007) as shown in (Figure 4).

#### 3.2.2. ECV Measured in the General Population in the Delayed Phase (DP) Setting

**Figure 5:**
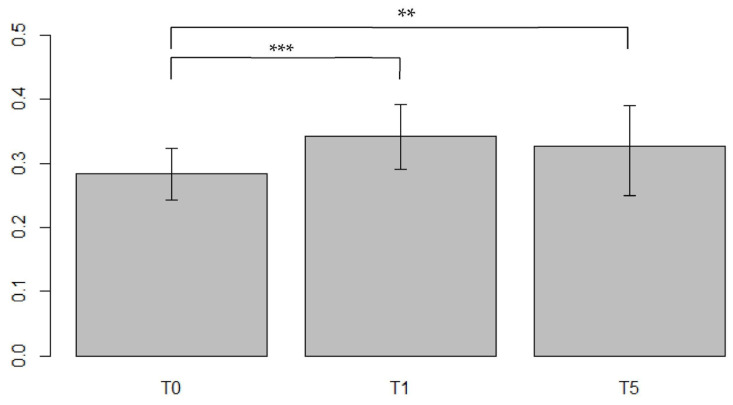
Comparisons of the ECV values from the same population between different timepoints in the Delayed Phase. Moreover, in Delayed Phase ECV values were measured at T_0_, T_1_ and T_5_. Both comparisons revealed values strongly significant (*p* < 0.001) confirming a strict interaction between CTX drugs and myocardial precocious damage. *: barely significant, **: significant, ***: strongly significant.

A positive result among the three populations (F = 5.6, *p* = 0.005) was found the ANOVA test. Afterward, a one-to-one subgroup analysis was performed analyzing the differences between the T_0_–T_1_ and T_0_–T_5_. The ECV values were compared between T_0_–T_1_ showing an RI of 20.6% (t_40_ = −3.9, *p* = 0.0003) and a significant increase of 15.6% between T_0_–T_5_ (t_40_ = −3.3, *p* = 0.002) (Figure 5).

### 3.3. Results for Doxorubicin Treated (DOX-Treated) Women in the PP and DP Settings

**Figure 6:**
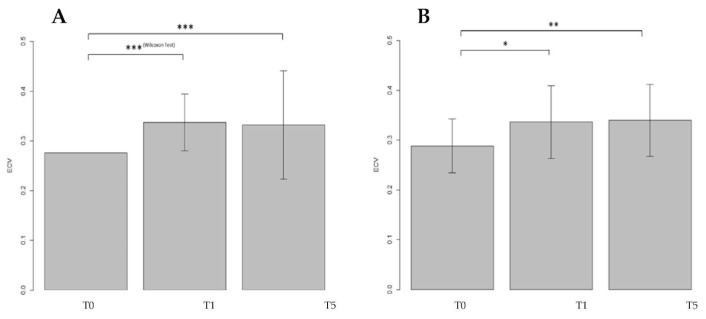
ECV mean values are presented in bar plots, while SD was expressed with error bars, at different FU times: baseline (T_0_), first year (T_1_) and fifth year (T_5_), both in portal phase (**A**) and in delayed phase (**B**) in patients treated with doxorubicin (DOX). Of note, T_0_ in PP was not normally distributed so SD was not used and the error bar was consequently not reported. A significant increase in mean values was found between T_0_–T_1_ and T_0_–T_5_. *** *p* < 0.001; ** *p* < 0.01; * *p* < 0.05.

A total of 54 patients were treated with doxorubicin, and all the patients completed the treatment. During the PP setting, ECV was measured at the baseline, T_0_ = 27.2% ± 10% (median ± IQR) and at the first year of FU, T_1_ = 34% ± 6% (Median ± IQR), with a relative difference (RD) of 22.4%, a value strongly significant (V = 342, *p* < 0.0001). Data were confirmed also at five years of FU (T_5_ = 31.4% ± 4%) compared to baseline with a relative reduction (RD) highly significant (RD = 14.0%, t_22_ = −9.00, *p* < 0.0001) (Figure 6A).

The cardiac ECV was measured also during the delayed phase (DP) setting, at the baseline (T_0_ = 28.4% ± 5%), at the first year of FU (T_1_ = 32.9% ± 7%), and at five years of FU (T_5_ = 33.3% ± 7%). Among these three groups, compared with each other, a statistically significant different was found (F_2.78_ = 5.57, *p* = 0.005) and the post hoc subgroup analyses revealed a significant increased cardiac ECV between T_0_–T_1_ (RD = 15.9%, t_26_ = 2.52, *p* = 0.018) and between T_0_–T_5_ (RD = 17.4% t_26_ = −3.03, *p* = 0.005), respectively (Figure 6B).

### 3.4. Results for Myocardial ECV in Patients Treated with EPI–TRAS Women in the PP and DP Setting

A total of 28 WB-CT examinations of EPI–TRAS treated BC women were included in the analyses. All the women completed successfully all the chemotherapeutic cycles. Similarly to DOX, cardiac ECV was measured also for EPI–TRAS treated women at T_0_ (26.7% ± 4%), at T_1_ (32.7% ± 4%) and at T_5_ (29.7% ± 4%), in PP setting. The global comparison with ANOVA test revealed a statistically significance among groups (F_2.39_ = 4.6, *p* = 0.016).

**Figure 7:**
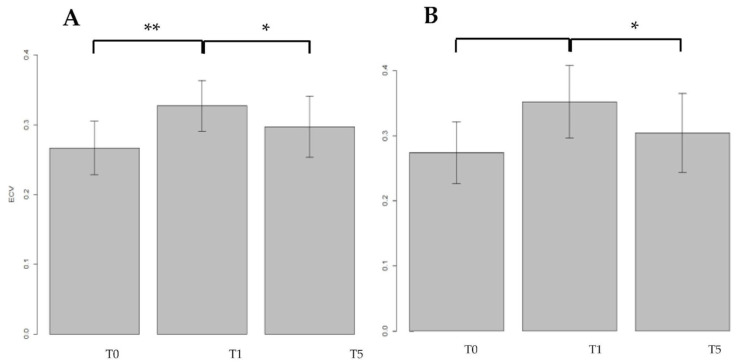
ECV mean values are presented in barplots at different FU times, at baseline (T_0_), at first year (T_1_) and at fifth year (T_5_), both in portal phase (**A**) and in delayed phase (**B**) in patients treated with epirubicin and trastuzumab (EPI–TRAS). *** *p*< 0.001; ** *p*< 0.01; * *p*< 0.05.

At the post hoc subgroup analyses we observed in the PP setting a significant increase of cardiac ECV from baseline to first year FU, with a significant RD between T_0_–T_1_ of 18.5% (t_13_ = 4.14, *p* = 0.001), but the result was not confirmed at the fifth year of FU (T_0_–T_5_), with an RD of 10.3% not reaching the significance (t_13_ = 1.66, *p* = 0.12). However, a significant reduction of ECV values was observed between T_1_–T_5_ with a significant RD of −10% (t_13_ = −2.23, *p* = 0.043) (Figure 7A).

The same 28 patients were examined after 5 min in DP setting, acquiring the images at T_0_ (27.4% ± 5%), at T_1_ (32.5% ± 6%) and at T_5_ (30.4% ± 6%). The comparison among the three temporal groups was statistically significant (F_2.78_ = 5.27; *p* = 0.009), so we could proceed with the post hoc subgroup analyses, which evidenced a significant increase of 28.7% between the T_0_–T_1_ (t_13_ = 3.27, *p* = 0.006) and a RD of −13.6% between T_1_–T_5_ (t_13_ = −2.36, *p* = 0.034), respectively (Figure 7B). Moreover, in this case, no differences were observed in cardiac ECV between T_0_–T_5_ (t_13_ = 1.60, *p* = 0.13).

### 3.5. Results for Myocardial ECV in Patients Treated with DOX versus Patients Treated with EPI–TRAS

**Figure 8:**
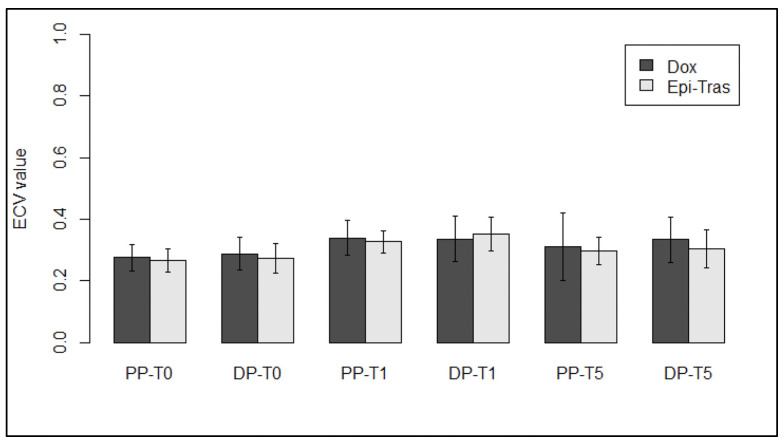
ECV comparison in patients treated with DOX versus patients treated with EPI–TRAS, at different FU times in both PP and DP setting. NS, not significant; PP, portal phase; DP, delayed phase.

At T_0_, no difference in the measured ECV values was observed between DOX- and EPI–TRAS-treated patients neither in PP (*p* = 0.51) nor in DP setting (*p* = 0.39), confirming the absence of cardiac damage before starting the NACT. However, in the PP, no difference was observed between the two different treated groups at T_1_ (V = 210, *p* = 0.57), and a similar result was achieved even at T_5_ (t_35_ = 1.07, *p* = 0.29). On the other hand, also in the DP setting between the two schemes, not statistically significant difference was observed neither at T_1_ (*p* = 0.48) nor at T_5_ (*p* = 0.10), respectively (Figure 8).

### 3.6. Clinical Assessments and CTRCD Events in the Treated Population

In our 82 patients undergoing cardiotoxic chemotherapy, echocardiography was performed at T_0_, T_1_= 12 m ± 3 m and T_5_ = 60 m ± 6 m with LVEF values at T_0_ (64% ± 5%), T_1_ (54% ± 6%) and T_5_ (53% ± 8%).

Among the selected 82 patients, we recorded 4 cases of cancer therapy-related cardiac dysfunction (CTRCD) during the period of follow-up from the end of the treatment (EOT). Indeed, we observed 2 patients suffering of LVEF reduction more than 15% after 16 and 23 months from EOT, respectively, 1 patient reported incidence of atrial fibrillation and pericardial effusion 14 months after EOT, and 1 patient reported an asymptomatic decrease of LVEF under the value of 50% (LVEF 40%) 16 months after EOT. In this subgroup, ECV measured in T_1_ and T_5_ was increased if compared to the baseline values (T_0_ = 0.26, T_1_ = 0.34, T_5_= 0.39).

## 4. Discussion

Our investigation is the first study, to our knowledge, comparing DOX with the EPI–TRAS combination using ECV values as an imaging marker measured from a WB-CT scan as source. This marker was already proven to be effective in the particular context of cardiac MRI and CT scans [7,13,16] in the acute and chronic CTX settings; in our case, however, the cost-effectiveness and routinary use of WB-CT in an oncological clinical setting is the major strength point of this study. Furthermore, it is the first study with a long-lasting FU with a timepoint assessed at five years from chemotherapy termination, whereas the other authors reported last evaluations at the latest within the first 2 years [13,22,23].

Classical biochemical markers such as troponin I (cTnI), troponin T (cTnT) and Natriurec Peptides (NP) have important limitations such as assay selection, different laboratory methods and cut-off values used, the variable follow-up and the lack of standardized cardiac endpoints [27,28] resulting in low sensitivity and serious difficulties to predict early CTX. For this issue, new biomarkers are under investigation, such as MPO [29], miRNA and circRNA [30], but ECV seems to be a promising reliable diagnostic marker for impending CTX.

Our results showed ECV values increasing more than 30% in T_1_ and T_5,_ whereas LVEF showed a slight decrease, without falling below the threshold value (LVEF < 50%).

Cardiac ECV measured from WB-CT scans can be used as early marker of myocardial damage in BC patients undergoing cardiotoxic chemotherapies, showing different kinetics for acute (T_1_) and for chronic settings (T_5_) and between PP an DP. In these settings, we observed in a court of 82 patients a general increase in ECV values, significantly different between pre- and post-NACT, within the first year (T_0_–T_1_) and the fifth year (T_0_–T_5_). An accepted explanation is the subclinical damage caused from the antiblastic toxicants generating a tissue oedema which is eventually evolved in fibrosis, as also described by other authors [31,32,33].

The slight difference in data obtained in portal or delayed phase (1 or 5 min) can be partially explained by different contrast permeability over time, inter-individual physiological or paraphysiological differences. Nevertheless, methodological issues should be taken into account; we conducted a non-randomized and non-matched retrospective study.

The AC induced CTX is widely described as cumulative and dose-related cardiotoxicity causing phenomena such as arrhythmias, ischemic disease, prolonged QT interval, valve dysfunction and systolic dysfuction [34]. Trastuzumab is not dose dependent, is often reversible and it is linked with systolic dysfunction, hypertension and, in rare cases, to ischemic disease [35]. On this topic, an old classification could explain the damage mechanisms, based on Type I and Type II CTX [36]. Indeed, the former is a typical anthracycline-related adverse event, it is dose-dependent and it is responsible for myocardial necrosis, vacuolation and oxydative stress. Furthermore, despite DOX being an effective drug in BC treatment, it shows an increased risk of CTX above the cumulative dose of 250 mg/m^2^ and reaches an unacceptable threshold at 550 mg/m^2^, whereas epirubicin showed comparable cumulative thresholds at 360 mg/m^2^ and 900 mg/m^2^, respectively [37,38]. Contrarily, the type II damage is typical of trastuzumab, which is not dose-related and classically considered recoverable, although recent data showed it to have a variable clinical outcome [36,39,40].

DOX and EPI–TRAS showed two different damage patterns associated with different modifications in ECV values at the different timepoints (T_0_, T_1_, T_5_) and at different phases (PP, DP) in BC patients. Indeed, in the DOX group, we observed concordant increasing ECV values at the first year for PP (T_0_–T_1_ 27.6% ± 5% vs. 34% ± 6%, RD of 22%, *p* < 0.0001) and for DP (T_0_–T_5_ 28.4% ± 5% vs. 32.9% ± 7%, RD 16%, *p* = 0.018), respectively. Noteworthy, the values remained stably high at T_5_ with 31.4% ± 4% with an RD from baseline of 14% (*p* < 0.0001) and 33.3% ± 7% with a RD of 17% (*p* = 0.005) for PP and DP settings, respectively (Figure 6). Our results are largely comparable with others in the literature [7]. Monti et al. described similar results although FU results were not significant when compared to baseline levels, probably because the timepoint of FU assessment was too early (median FU 135 days). Conversely, our work, focusing on longer FU, found significant differences and probably allows for a better distinction between the acute from the chronic damage [7]. As expected, this phenomenon is due to the type I cardiac damage, radiologically confirming that DOX could cause a subclinical cardiac damage, in the absence of clinical signs, that persists over time and could eventually evolve in CTX as well [13]. Moreover, the study by Monti et al. was focused on a population treated with epirubicin only, whereas our study was based on the direct comparison between DOX vs. EPI–TRAS for longer FU. Our data from EPI–TRAS highlighted a maximum peak of ECV values at T_1_ (32.7% ± 4%) vs. baseline (T_0_ = 26.7% ± 4%) detecting a RD of 18% for PP (*p* = 0.001), whereas a RD of 29% (32.9% ± 7% vs. 28.4% ± 5%, *p* = 0.006) was found in DP. However, in both phases, a drop was observed almost to baseline values at T_5_ (T_0_–T_5_ lapse *p* = 0.012 and *p* = 0.13 for PP an DP, respectively). The decreasing ECV values in T_1_–T_5_ lapse were statistically significant both in PP (RD −10.0%, *p* = 0.043) and in DP (RD −13.6% *p* = 0.034, Figure 7). This trend in the general asymptomatic population exposed to EPI–TRAS suggests a recover from an early damage in agreement with the type II damage. This effect is largely attributable to trastuzumab-related cardiac effect [39,41], whereas epirubicin is known to have fewer cardiotoxic effects than DOX. Indeed, at the cumulative dose of 900 mg/m^2^, epirubicin is responsible for an incidence peak of only 3.3% and the risk of HF become consistent only when exceeding this threshold [37,38,41]. Of note in our study, epirubicin was delivered at 300 mg/mq, far lower than the threshold previously described, thus confirming trastuzumab as the relevant cardiac toxicant. Our observation is confirmed by other authors who failed to find a statistically significant difference in ECV values from baseline in women treated with schemes with only epirubicin as the cardiac toxicant at the same doses [7]. To our knowledge, we are the first to describe this phenomenon at 5 years [39,41,42], confirming a possible recovery from EPI–TRAS subclinical damage.

No statistical difference was found between the DOX and EPI–TRAS schemes, although a non-significant trend was found at the fifth year of FU (Figure 8). However, nowadays the type I and type II CTX distinction is considered old and imprecise due to the fact that the two phenomena could intertwine, in particular, because AC and trastuzumab can be used in combination. Indeed, the incidence of heart failure was found to attain around 5% from old studies (4% for NHYA class III/IV) when trastuzumab was sequentially administered after anthracyclines as stated by the “double hit” theory [36]. In any case, a recent prospective study, the CARDIOTOX trial, using updated definitions of CTX, followed 148 patients who received AC + trastuzumab, for a median period of 24.1 months and CTX events were assessed to be 38.6% for mild and 6.4% for moderate symptoms, whereas only 0.7% of patients experienced more serious cardiac events such as heart failure (HF) [42]. Our results mainly describe a population comparable with the mild to moderate CARDIOTOX trial population, giving interesting evidence about cardiac modification detectable in WB-CT scans. These findings could be useful in further randomized, controlled studies in order to assess early CTX in addition to the standard diagnostic tools as serum biomarkers and echocardiograms.

An unmodifiable bias was represented by the combination of drugs known to have a cardiotoxic effect such as ischemic disease (5-Fluoruracil, Cyclofosphamide), arrhytmias (Taxanes), LVEF depression (5-Fluoruracil) used in polychemotherapeutic schemes, in agreement with current guidelines.

Another important limitation was that the chosen hematocrits were performed in a different moment from the WB-CT, but still no more than 4 weeks before or after the imaging acquisition. Little influence on intra-individual variability was expected, as is analogous to other authors as described in the relevant literature [13].

The main limitation of this study remains the low diagnostic accuracy, due to the lack of ECG gating scans, leading to possible motion artifacts and intra-individual variability. Nevertheless, this is also a strength due to the attempt to simplify a cumbersome investigation in order to use it as a possible clinical routine method. In the attempt to raise accuracy, an interesting perspective could be performing an additional arterial-phase scan with a restricted field of view (FOV) over the heart in follow-up studies of selected patients at high risk of developing CTX. Although a minimal increase in radiation dose is expected in this way a greater accuracy in CTX diagnosis would be warranted.

However, after a possible validation, WB-CT could be used for CTX screening, while ECG gated CT scans, additional FOV heart-centered arterial phase or cardiac MRI could be reserved for a further selected investigation.

## 5. Conclusions

We determined that BC women of age 55 ± 9 years who underwent different schemes of NACT expressed different ECV values at CT scans during FU. Our study has confirmed that myocardial ECV can be effectively calculated from CT scans [13,21,22], being an easily obtainable and reproducible measurement. Therefore, CT-derived ECV could be a valid imaging marker for the detection of myocardial damage and prevention of cardiotoxicity in patients undergoing cancer therapy. Starting from our evidence, more studies are needed to show the efficacy of this marker in WB-CT scans. Indeed, larger prospective trials are needed to validate these promising data based on WB-CT, largely used by clinicians, in order to detect early cardiotoxicities. These would help distinguish type I from type II damage and explain the development of severe CTX linked with AC and trastuzumab use.

## Figures and Tables

**Table 1:** Main characteristics of the study population.

Variables	Value (*n* = 82)
Age (years), mean ± DS	54.9 ± 5
Type of carcinoma	
Infiltrating ductal	62
Infiltrating lobular	4
Poorly differentiated	14
Undifferentiated	2
Disease stage (TNM)	
Ic	8
II	44
III	14
IV	16
Treated with doxorubicin	54
epirubicin-trastuzumab	28
LVEF (%), mean ± SD	64% ± 5%
Hematocrit (%), mean ± SD	36.8% ± 4%

SD, standard deviation; TNM, tumor node metastasis; LVEF, left ventricular ejection fraction.

**Table 2:** Inter-observer differences and reproducibility between operators in Portal Phase and Delayed Phase.

**Portal phase**	**Operator 1** **Mean (SD)**	**Operator 2** **Mean (SD)**	***p*-value**
Baseline (T_0_)	0.270 ^#^ (0.06) ^#^	0.290 ^#^ (0.08) ^#^	0.09 ^#^
1st year FU (T_1_)	0.326 (0.06)	0.329 (0.05)	0.77
5th year FU (T_5_)	0.300 ^#^ (0.05) ^#^	0.310 ^#^ (0.07) ^#^	0.68 ^#^
**Delayed Phase**	**Operator 1** **Mean (SD)**	**Operator 2** **Mean (SD)**	***p*** **-value** ^**#**^
Baseline (T_0_)	0.283 (0.05)	0.279 (0.06)	0.64
1st year FU (T_1_)	0.345 (0.07)	0.335 (0.06)	0.47
5th year FU (T_5_)	0.327 ^#^ (0.07) ^#^	0.312 ^#^ (0.07) ^#^	0.34 ^#^

FU, follow-up, SD, standard deviation. ^#^ for baseline of portal phase and fifth year of portal and delayed phase a not normal distribution was found. For this reason, the wilcoxon–mann–whitney test and median + IQR as point estimators were used.

## Data Availability

The datasets used and analyzed during the current study are available from the corresponding author on a reasonable request.
